# Supplementary material for: Influenza A Virus Infection Reactivates Human Endogenous Retroviruses Associated with Modulation of Antiviral Immunity
Source: Viruses. 2022 Jul 21;14(7):1591. doi: 10.3390/v14071591 (PMC9320126; doi:10.3390/v14071591)
Supplement: Supplementary file 1 [file viruses-14-01591-s001.zip › MotifAnalysis.zip!/MotifOutput_SC35MdNS1/homerResults/motif14.similar.html]

motif14

## Information for motif14

G
T
A
C
A
C
T
G
A
C
T
G
C
T
G
A
A
G
T
C
A
G
T
C
C
G
T
A
A
C
T
G
A
C
T
G
C
G
T
A
C
G
T
A
C
G
T
A
  
Reverse Opposite:  

A
C
G
T
A
C
G
T
A
C
G
T
A
G
T
C
A
G
T
C
A
C
G
T
A
C
T
G
A
C
T
G
A
G
C
T
A
G
T
C
A
G
T
C
A
C
T
G
  

|  |  |
| --- | --- |
| p-value: | 1e-17 |
| log p-value: | -3.981e+01 |
| Information Content per bp: | 1.956 |
| Number of Target Sequences with motif | 7.0 |
| Percentage of Target Sequences with motif | 9.09% |
| Number of Background Sequences with motif | 7.9 |
| Percentage of Background Sequences with motif | 0.02% |
| Average Position of motif in Targets | 50.6 +/- 46.7bp |
| Average Position of motif in Background | 77.5 +/- 45.1bp |
| Strand Bias (log2 ratio + to - strand density) | 10.0 |
| Multiplicity (# of sites on avg that occur together) | 1.00 |
| Motif File: | file (matrix) reverse opposite |

### Similar de novo motifs found

|  |  |  |  |  |  |  |  |
| --- | --- | --- | --- | --- | --- | --- | --- |
| Rank | Match Score | Redundant Motif | P-value | log P-value | % of Targets | % of Background | Motif file |
| 1 | 0.840 | G T C A C T A G C T A G C T G A A G T C A G T C C G T A T A C G A C T G C T G A | 1e-10 | -23.576315 | 10.39% | 0.28% | motif file (matrix) |
| 2 | 0.666 | T A C G A C G T A G T C A G T C C G T A A C T G A C T G C G T A A G T C C G T A | 1e-5 | -12.623250 | 6.49% | 0.29% | motif file (matrix) |
| 3 | 0.623 | C G T A A G T C A G T C C G A T A C T G A C T G A G C T C G T A | 1e-4 | -9.890566 | 12.99% | 2.75% | motif file (matrix) |
| 4 | 0.682 | A C G T A G T C A G T C C G A T A C T G A C T G C G T A A G T C | 1e-4 | -9.497905 | 9.09% | 1.32% | motif file (matrix) |
